# Real-time ultrasound for tip location of umbilical venous catheter in neonates: a pre/post intervention study

**DOI:** 10.1186/s13052-021-01014-7

**Published:** 2021-03-18

**Authors:** Serena Antonia Rubortone, Simonetta Costa, Alessandro Perri, Vito D’Andrea, Giovanni Vento, Giovanni Barone

**Affiliations:** 1 Department of Woman and Child Health and Public Health, Division of Neonatology, University Hospital Fondazione Policlinico Gemelli IRCCS, Rome, Italy; 2 Neonatal Intensive Care Unit, Infermi Hospital, Rimini, AUSL of Romagna Italy

**Keywords:** Neonates, Real-time ultrasound, Training, Umbilical venous catheter

## Abstract

**Background:**

Recent guidelines advocate the use of real-time ultrasound to locate umbilical venous catheter tip. So far, training programs are not well established.

**Methods:**

A pre/post interventional study was carried out in our tertiary neonatal intensive care unit centre to evaluate the efficacy of a training protocol in the use of real-time ultrasound. Primary outcome was the percentage in the use of real-time ultrasound.

**Results:**

Fifty-four patients were enrolled. The use of real-time ultrasound for tip location significantly increased after the training program (15.3% vs 89.2%, *p* <  0.0001). After the training the tip of the catheters was more frequently placed at the junction of the inferior vena cava and right atrium (75% vs 30.7%, *p* = 0.0023). Twenty-two catheters were also evaluated with serial scans during the intervention phase to assess migration rate which was 50%.

**Conclusion:**

a multimodal, targeted training on the use of real-time ultrasound for umbilical venous catheter placement is feasible. Real-time ultrasound is easily teachable, increases the number of umbilical venous catheters placed in a correct position, reduces the number of line manipulations and the need of chest-x-rays.

**Supplementary Information:**

The online version contains supplementary material available at 10.1186/s13052-021-01014-7.

## Introduction

The umbilical venous catheter (UVC) is currently one of the most common central venous access devices used in neonatal intensive care unit (NICU). It is very easy to place, more stable when compared to a peripheral venous line and suitable for preterm and critically ill term infants who require fluids, inotropes, parenteral nutrition or frequent blood sampling [1]. The UVC is usually inserted by skilled medical staff at a distance previously calculated using anthropometric measures or formulas and nomograms usually based on birth weight (BW) [2, 3].

The ideal UVC tip position is outside the heart at the junction of inferior vena cava (IVC) and right atrium (RA) [1, 4–7]. This position has been associated with less incidence of early and belated life-threatening complications such as pericardial and pleural effusion, cardiac tamponade, endocarditis, cardiac arrhythmias, liver haematoma, necrosis or other parenchymal injuries, necrotizing enterocolitis, thrombosis and portal hypertension [4, 8–10].

Antero-posterior chest radiography (CR) is the most commonly used method to locate catheter tip [11, 12] using as landmarks either the thoracic vertebral bodies or cardiac silhouette although a significant number of studies has questioned the accuracy of CR for this purpose [13–17].

On the other hand ultrasonography (US) has been suggested in several papers as gold standard [13–17] as it seems more reliable, faster and without side effects [13–16, 18–22]. US can be performed at the bedside during the insertion procedure (real-time US,) avoiding multiple catheter manipulations and allowing an immediate and safe injection of fluids and medications [7, 15, 18].

US also allows monitoring the UVC tip position over time, since tip migration has been described up to 50–90% of the cases [22, 23].

Despite all these proven advantages, US is still not widely used in many NICUs to assess the UVC tip position, possibly because it needs additional and adequate training of the medical staff. In many studies scan was performed by paediatric radiologists or cardiologists [14, 16, 19, 24, 25] that are not always available or on-call, but recent studies have suggested that training on the use of real-time US (RUS) is easy and feasible [15, 17, 25–28].

In our Neonatal Unit, US is widely used by neonatologists and neonatal fellows: many of them are able to perform head US, echocardiography and lung US. Nevertheless US is not always routinely used for intraprocedural assessment of UVC tip position since not the whole medical staff is completely trained and skilled to it.

This pre/post-intervention study was designed to evaluate the feasibility of a basic, multimodal training of the medical staff, with the aim to implement the use of RUS to localize the UVC tip position.

## Materials and methods

### Study design, setting and patient population

This was a pre/post-intervention study.

The study was conducted at the University Hospital “A. Gemelli” of Rome between January and May 2018. The institutional review board approved the study. Parental written consent was obtained for all the patients included in the study, specifying the need to perform repeated US, only on babies in stable conditions.

All inborn infants admitted to our Neonatal Wards (NICU, Neonatal high-dependency and Special Care Units) who required UVC placement were eligible for the study. Exclusion criteria included infants who needed resuscitation in delivery room with medications or fluids infused via UVC and babies with major congenital anomalies.

UVCs were placed by neonatologists or neonatal fellows under sterile condition using standard clinical techniques. The estimated length of the UVC insertion was calculated as BW (g) × 0.5 + 5.6 cm [29]. Single or double lumen catheters with diameters ranging from 3.5 to 5 Fr were chosen depending on the infant’s BW and clinical conditions, according to our local guidelines [30]. The decision regarding which method (CR or RUS) should be used to assess the catheter tip was left to the attending neonatologist during all the phases of the study. Catheters were sutured on Wharton jelly and then taped on the skin with steri-strips and covered with transparent film dressing.

### Study phases

The study was set in three different phases: pre-intervention, training (intervention) and post-intervention phase.

### Pre-intervention phase

During the first phase (January – February 2018) the total number of UVC positioned, the method used to localize the tip (CR or US), the total number of CR required for UVC adjustments to obtain the final correct position, the time to have radiographic images available, catheter related complications and the incidence of sepsis were prospectively recorded. Clinical information collected included gestational age (GA), BW, sex, mode of delivery, antenatal steroids, resuscitation in delivery room, apgar score.

### Training phase

March 2018 was the intervention (training) month. All the medical staff was addressed and invited to participate to a training program on US-guided UVC placement.

The primary goal of this project was to create the awareness that a relatively simple and easy-to-learn technique could improve the quality of our routine clinical practice, according to literature evidences and guidelines.

The training was held by a neonatologist (GB) and a neonatal fellow (SAR) who were already trained and experienced in using US for UVC positioning, since they attended certified courses to perform neonatal echocardiography (Echo-team).

### US-guided UVC placement

UVC was inserted under sterile condition using standard clinical procedure previously described in this article. A S4–10 Ultrasound Broad Spectrum sector transducer set at 7 MHz (GE Healthcare, Little Chalfont, United Kingdom) was connected to a LOGIQ E9 Ultrasound Unit (GE Healthcare, Little Chalfont, United Kingdom) for ultrasound imaging. Maximal barrier precautions were used, including covering the probe with a long sterile sheath and using sterile gel. Two operators were usually involved in the procedure, one placing the catheter and the other performing RUS in order to decrease the time needed for the procedure.

**Fig. 1:**
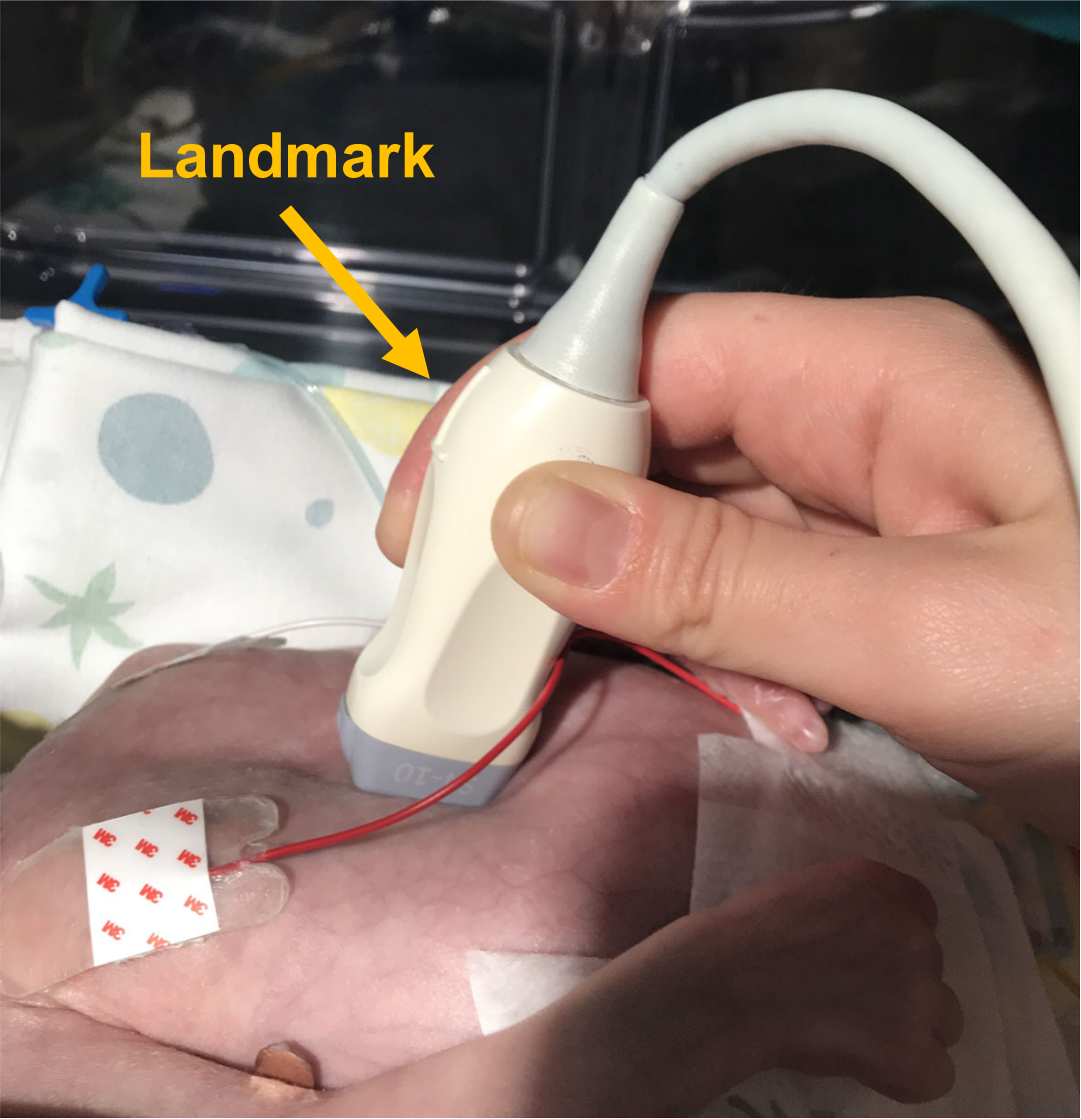
Probe position (landmark towards the baby’s head) to obtain the parasagittal sub-xiphoid view

**Fig. 2:**
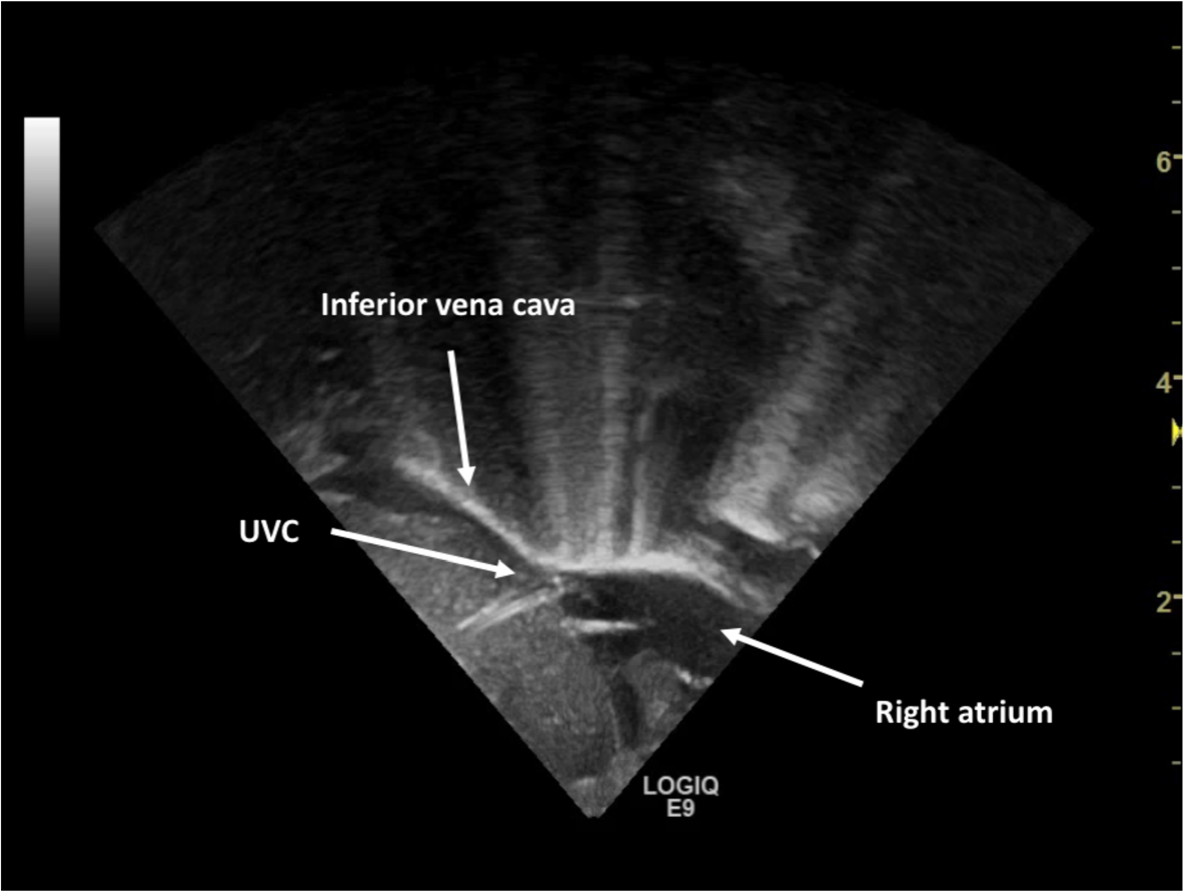
Ultrasound scan of the correct UVC tip position at the junction of inferior vena cava-right atrium

The US operator placed the probe over the mid-chest with a parasagittal sub-xiphoid view (Fig. 1) to identify anatomic structures (Fig. 2). After catheter insertion US was used to perform tip navigation following the catheter into the IVC. Tip was then located at the IVC-RA junction. The injection of a small volume (0.5–2 mls) of saline was used to confirm the tip position [see the video as Additional file]. When the team was satisfied with the final tip position, the catheter was secured, taped and covered. If the UVC tip was not surely identified during the US-guided procedure the decision to perform a CR was left to the attending neonatologist.

The Echo-team always supervised and guided the procedure during the training phase. All UVCs correctly positioned in babies in stable conditions were re-checked in order to give the clinicians several chances to look at the tip location.

The training program was multimodal and conducted by mean of formal classroom lessons and both collective or one-on-one bedside teaching and practice. Informative didactic material was available for the trainees in all the neonatal wards (Table 1).

**Table 1:** Training Program

**Formal classroom lesson**	**Duration** 40 min **Timing** Repeated weekly (4 times in a month) to allow all the trainees to attend at least once. **Aims** Showing and highlighting the advantages of using RUS vs traditional chest radiography for UVC insertion and localization, according to literature. Teaching how to perform an US-guided UVC placement. **Materials and Methods** Case reports and images from literature and clinical practice on correct and incorrect UVC positions. Images and descriptions of all the potential complication of a malpositioned UVC. Images and US scans of anatomic and vascular structures to be identified for the procedure. US scans (images and videos) of UVC in correct or incorrect positions. Description, images and videos of how to perform the complete US-guided UVC positioning procedure.
**Bedside US practise**	**Timing** After each frontal lesson and anytime trainees were interested in practising US on babies with an UVC in the correct position, only when in good and stable conditions (parental written consent was previously obtained) **Aims** Showing how to set the bedside US and the correct probe. Teaching how to identify anatomic and vascular structures and the catheter tip position in babies with an UVC in a good position (parental consent was previously obtained). **Methods** The Echo-team member assisted as a direct supervisor a trainee while performing the scans.
**Bedside teaching**	**Timing** Every time (daytime) an UVC was placed during the training period **Aim** **T**eaching how to perform a complete US-guided UVC placement **Methods** The Echo-team member performed the RUS or assisted as a supervisor a trainee in the US-guided UVC placement.
**Informative teaching material**	**Aim** Improving access to the training program and contents and support the learning. **Materials and Methods** Posters containing description and illustrations of the entire procedure were posted on walls in each ward and pocket cards were available for all the trainees. They explained how to set the US machine, which probe to use, where to find all the materials, how to set the sterile field with the probe. The complete sequence of the US-guided UVC placement was described and explanatory images showed.
**Weekly surveys**	**Aim** Improving or changing some aspects of the training. **Materials and Methods** Written questionnaires or talk with a Echo-team member through which the trainees were frequently encouraged to express their approval or concerns.

### Post-intervention phase

In the 2 months following the training (April – May 2018) the same information collected in the pre-intervention phase were recorded.

All UVCs placed during this study phase by the trained medical staff were double-checked by the Echo-team (as soon as possible after the positioning) in order to verify skills and avoid unsafe positions.

Moreover all the catheters placed at the IVC-RA junction were visualized by US within 24 h from the positioning (day 0) and then over time on day 1, 3, 5 and 7 from the insertion to evaluate catheter migration. All the US to assess migration were performed by the same operator (SAR).

### Outcome

Our primary outcome was the percentage in the use of RUS for UVC tip location during pre and post- intervention phases.

Secondary outcomes included the proportion of UVC in a correct position after insertion, the incidence of catheter-related complications, the number of UVC manipulations in the first 24 h, the number of CR to visualize and/or to make adjustments of the catheter tip, UVC dwell time and the percentage of catheter migration.

### Statistical analysis

Statistical analyses were performed with Microsoft Excel 2003 (Microsoft, Redmond, WA) and SPSS for Windows 17.0 (SPSS, Chicago, IL). We used Student’s t-test for normally distributed continuous variables and Fisher’s exact test for categorical variables. The Wilcoxon rank-sum test was used to analyse differences in continuous variables, which were not normally distributed. *P* values of 0.05 were considered to be statistically significant.

## Results

Among the consultants and trainees 53 out of 67 (79.1%) doctors attended the training.

**Fig. 3:**
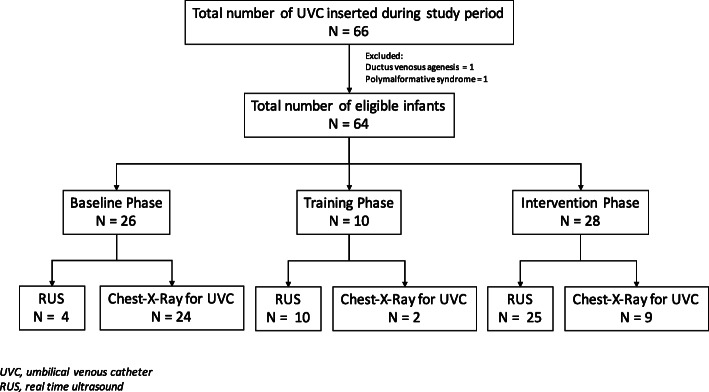
Flow diagram of the study population

Sixty-six patients underwent umbilical venous catheterisation during the study period, 54 were enrolled and analysed: 26 infants in the pre-intervention phase and 28 infants in the post-intervention phase (Fig. 3).

The reasons for UVC positioning were the following: in the pre-intervention phase 7 babies needed an UVC for prematurity, 6 for birth asphyxia, 5 for respiratory distress syndrome (RDS), 3 for surgical conditions, 4 had intrauterine growth restriction (IUGR) with abnormal antenatal doppler, 1 had a cardiac arrhythmia, in the post-intervention phase 3 neonates needed UVC for prematurity, 5 for birth asphyxia, 13 for RDS, 1 for surgical condition, 4 for IUGR with abnormal antenatal doppler, 1 for suspected sepsis, 1 for isoimmunization.

Baseline characteristics of infants included in the study are listed in Table 2.

**Table 2:** Baseline characteristics of infants in the pre and post-intervention phases. Data are shown as number (%), median (interquartile range) or mean (standard deviation)

Gestational age (wks) 29–36	11 (42.3)	16 (57.1)	0.06
Gestational age (wks) 37–42	9 (34.6)	9 (32.2)	0.44
Birth weight (g)	1808 [1060–3106]	2305 [1488–3110]	0.2775
VLBW	13 (50.0)	8 (28.6)	0.15
AGA	19 (73.0)	19 (67.8)	0.7698
Male	18 (69.2)	18 (64.2)	0.7773
Twins	3 (11.5)	3 (10.7)	1.0000
Cesarean section	22 (84.6)	18 (64.2)	0.4896
Abnormal antenatal Doppler	3 (11.5)	6 (21.4)	0.4704
Resuscitation in delivery room	21 (80.7)	20 (71.4)	0.5303
Apgar 1 min	6 [5–8]	8 [5–8]	0.3454
Apgar 5 min	8 [7–9]	8 [8–9]	0.8490

The primary and secondary outcomes are reported in Table 3: the use of RUS for tip location significantly increased after the training while the use of CR decreased, as expected. The average time to visualize the position of the catheter tip did also differ between the two phases since it was significantly lower in the post-intervention phase. The number of UVC manipulations in the first 24 h was not significantly different, while the UVCs placed during the post-intervention phase were more frequently placed at the IVC-RA junction. There was no significant difference in the other secondary outcomes (Table 3).

**Table 3:** Primary and secondary outcomes. Data are shown as number (%) or median (interquartile range), mean (standard deviation)

	Pre-Intervention Phase *N* = 26	Post-Intervention Phase *N* = 28	*P*
Real-time US	4 (15.3)	25 (89.2)	< 0.0001
Chest X-Ray	24 (92.3)	9 (32.1)	< 0.0001
Time to visualize UVC tip (h)	2 [1–3]	0 [0–1]	0.0012
N of manipulations first 24 h	11 (42.3)	5 (17.8)	0.0740
UVC in a good position	8 (30.7)	21 (75.0)	0.0023
Double lumen UVC	21 (80.7)	21 (75.0)	0.7471
Size 3.5–4 Fr	18 (69.2)	16 (57.1)	0.4082
Size 5 Fr	8 (30.8)	12 (42.9)	0.4082
UVC dwell time (days)	4.7 (2.7)	5.4 (2.5)	0.3306
Sepsis	2 (7.7)	0	0.2271
Elective UVC removal	19 (73.1)	24 (85.7)	0.3198
Catheter-related complications	2 (7.7)	0	0.2271
Antibiotic therapy	20 (76.9)	20 (71.4)	0.3187

Twenty-two catheters located at the IVC-RA junction were evaluated over time with serial scans to study tip migration. Overall 11/22 infants (50%) experienced catheter migration. Ten catheters moved inwards into the right or left atrium and one moved outwards into hepatic vessels. The highest migration rates were registered on day 1 and day 3 (Table 4).

**Table 4:** Ultrasound findings of catheter tip position and migration rate over time. Data are shown as number (%)

	US on Day 0 (*N* = 22)	US on Day 1 (N = 22)	US on Day 3 (*N* = 19)	US on Day 5 (*N* = 17)	US on Day 7 (*N* = 11)
Good position	18 (81.8)	17 (77.3)	13 (68.4)	16 (94.1)	11 (100)
Malposition	4 (18.2)	5 (22.7)	6 (31.6)	1 (5.9)	–
Right atrium	3 (13.6)	4 (18.2)	4 (21.1)	1 (5.9)	–
Left atrium	1 (4.6)	–	–	–	–
Hepatic vessels	–	1 (4.5)	2 (10.5)^a^	–	–
Migration Rate	4 (18.2)	5 (22.7)	4 (21.1)	1 (5.9)	0

^a^Dislodgment

**Fig. 4:**
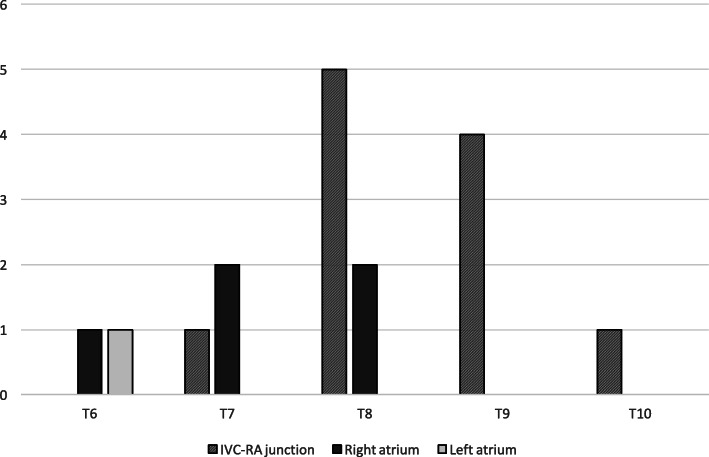
UVC tip position on ultrasound by vertebral body level

In 17 patients, who underwent CR for UVC tip location or for lung or abdominal disease, UVC tip position on US was compared to vertebral body (Fig. 4). As expected the location of UVC based on vertebral bodies proved to be unreliable [22].

## Discussion

A basic, targeted training on US-guided catheter insertion is effective in increasing the use of RUS and decreases the use of CR.

Physicians were able to learn the proposed technique for tip navigation and tip location after one theoretical lesson and multiple bedside practical sessions.

Our study demonstrates that a focused intervention, such as a targeted training, can increase awareness of the benefits and consequently implement the use of a technique that has already been validated as the gold standard for catheter tip location. To the best of our knowledge there are only few studies focusing on a well-established and specific training of the neonatal medical staff on the use of US in UVC line placing, one of which used an animal model [17, 31].

Of great interest in our paper is the reduction in the number of CR to visualize UVC. Several studies demonstrated that the use of lung US significantly reduced radiation exposure in preterm neonates admitted to NICU [32], and we speculate that the use of US-guided catheter procedures could further contribute to this purpose, especially in preterm babies which are likely the most vulnerable population with regard to the long-term effects of ionizing radiation.

Another interesting finding is that the use of RUS significantly reduced the time to visualize the UVC position and consequently the time needed to safely use this venous access device. According to the Infusion Nursing Society guidelines [7] clinicians have to verify tip location prior to start infusion. Infusion of high osmolality solutions like parenteral nutrition or medications like inotropes can be associated with severe complications in case of a high or low position of the catheter, so an immediate correct visualization of the UVC tip is crucial. Our study confirms that US is more accurate in regard to tip location when compared to CR. This finding is expected, in fact the location of the tip at x-ray is based on the relationship between the projection of the tip and some non-vascular radiological landmarks, such as the vertebral bodies and/or to the diaphragm. On the contrary, US can detect the position of the tip inside the vasculature with the best precision [15, 17, 24].

Migration of the UVC tip has also been documented and Franta et al. and Hoellering et al. recently demonstrated that it can occur up to 50% [22] or 90% [23] of the cases. It is commonly attributed to drying of the Wharton jelly and secondary shortening of the umbilical cord remnant. In our study half the infants experienced catheter migration. It occurred mainly inwards (in right or left atrium) between 24 and 48 h. We could not document any significant association with ventilation mode or other clinical parameters.

The execution of repeated US scans both for training purposes and to study catheter migration might create discomfort or instability, especially on preterm babies. Nevertheless we think that the discomfort and the stability of the patient must be balanced against the risk of malposition or migration of such catheters. Several reports [22, 23], including our findings, suggest that UVC migration is a quite frequent event. This is particularly important, especially in preterm infants, because migration and secondary malposition have been recently associated with the risk of NEC [33].

Considering our experience and previous results, we encourage the use of US not only for UVC placement but also for tip monitoring in order to avoid complications due to migration.

To the best of our knowledge this is the first study that completely focuses on and demonstrates the feasibility of a basic, multimodal medical staff training on the use of RUS for UVC localisation, with a significant impact on the routine clinical practice. Our targeted training protocol could be easily adopted by other tertiary care neonatal centres in order to achieve good results in terms of accuracy, reduction of radiation exposure and time-saving deriving from the use of RUS.

We believe that the following factors played a major role in achieving a positive outcome: identifying the problem and the incidence of catheter malposition, creating awareness of all the related complications, developing a targeted and basic training program, using multiple methods, easy-to-follow and with a reasonable duration, tracking the implementation process by weekly surveys among the trainees.

One limitation of this study is the relatively small number of patients. However, the sample size was enough to meet the study aims, particularly to demonstrate the feasibility of the training and to measure the increase in the use of RUS to assess UVC tips position.

## Conclusion

The introduction of a targeted training for the medical staff on the use of RUS in NICU to guide UVC placement is feasible and resulted in an implementation of this technique with a reduction in the use of CR in our neonatal unit. US-guided umbilical line placement is a technique easy to learn, thus we encourage the introduction of similar training protocols in neonatal units with the aim to adopt RUS. We further suggest regular US evaluation of UVC position since catheters migration occurs very often and it is potentially related to severe complications.

## Supplementary Information

**Additional file 1.**

## Data Availability

The datasets used and/or analysed during the current study are available from the corresponding author on reasonable request.

## Abbreviations

UVC: Umbilical venous catheter
NICU: Neonatal intensive care unit
BW: Birth weight
IVC: Inferior vena cava
RA: Right atrium
CR: Antero-posterior chest radiography
US: Ultrasonography
RUS: Real-time ultrasound
QI: Quality improvement
GA: Gestational age
RDS: Respiratory distress syndrome
IUGR: Intrauterine growth restriction
